# Effect of an electronic nicotine delivery device (*e*-Cigarette) on smoking reduction and cessation: a prospective 6-month pilot study

**DOI:** 10.1186/1471-2458-11-786

**Published:** 2011-10-11

**Authors:** Riccardo Polosa, Pasquale Caponnetto, Jaymin B Morjaria, Gabriella Papale, Davide Campagna, Cristina Russo

**Affiliations:** 1 Centro per la Prevenzione e Cura del Tabagismo (CPCT), Azienda Ospedaliero-Universitaria "Policlinico-Vittorio Emanuele", Università di Catania, Catania, Italy; 2 Institute of Internal Medicine, S. Marta Hospital, Azienda Ospedaliero-Universitaria "Policlinico-Vittorio Emanuele", Università di Catania, Catania, Italy; 3 IIR Division, School of Medicine, University of Southampton, Southampton General Hospital, Southampton SO16 6YD, UK

## Abstract

**Background:**

Cigarette smoking is a tough addiction to break. Therefore, improved approaches to smoking cessation are necessary. The electronic-cigarette (e-Cigarette), a battery-powered electronic nicotine delivery device (ENDD) resembling a cigarette, may help smokers to remain abstinent during their quit attempt or to reduce cigarette consumption. Efficacy and safety of these devices in long-term smoking cessation and/or smoking reduction studies have never been investigated.

**Methods:**

In this prospective proof-of-concept study we monitored possible modifications in smoking habits of 40 regular smokers (unwilling to quit) experimenting the 'Categoria' e-Cigarette with a focus on smoking reduction and smoking abstinence. Study participants were invited to attend a total of five study visits: at baseline, week-4, week-8, week-12 and week-24. Product use, number of cigarettes smoked, and exhaled carbon monoxide (eCO) levels were measured at each visit. Smoking reduction and abstinence rates were calculated. Adverse events and product preferences were also reviewed.

**Results:**

Sustained 50% reduction in the number of cig/day at week-24 was shown in 13/40(32.5%) participants; their median of 25 cigs/day decreasing to 6 cigs/day (p < 0.001). Sustained 80% reduction was shown in 5/40(12.5%) participants; their median of 30 cigs/day decreasing to 3 cigs/day (p = 0.043). Sustained smoking abstinence at week-24 was observed in 9/40(22.5%) participants, with 6/9 still using the e-Cigarette by the end of the study. Combined sustained 50% reduction and smoking abstinence was shown in 22/40 (55%) participants, with an overall 88% fall in cigs/day. Mouth (20.6%) and throat (32.4%) irritation, and dry cough (32.4%) were common, but diminished substantially by week-24. Overall, 2 to 3 cartridges/day were used throughout the study. Participants' perception and acceptance of the product was good.

**Conclusion:**

The use of e-Cigarette substantially decreased cigarette consumption without causing significant side effects in smokers not intending to quit (http://ClinicalTrials.gov number NCT01195597).

## Background

With well over one billion smokers' worldwide, cigarette smoking is a global epidemic that poses a substantial health burden and costs [1]. This is because cigarette smoke harms several organ systems of the human body, thus causing a broad range of diseases, many of which are fatal [2,3]. The risk of serious disease diminishes rapidly after quitting and life-long abstinence is known to reduce the risk of lung cancer, heart disease, strokes, chronic lung disease and other cancers [4,5].

Although evidence-based recommendations indicate that smoking cessation programs are useful in helping smokers to quit [6], smoking is a very difficult addiction to break. It has been shown that approximately 80% of smokers who attempt to quit on their own, relapse within the first month of abstinence and only about 3-5% remain abstinent at 6 months [7]. Although there is little doubt that currently-marketed smoking cessation products increase the chance of committed smokers to stop smoking, they reportedly lack high levels of efficacy, especially in the real life setting [8]. Although this is known to reflect the chronic relapsing nature of tobacco dependence, the need for novel and effective approaches to smoking cessation interventions is beyond doubt.

**Figure 1:**
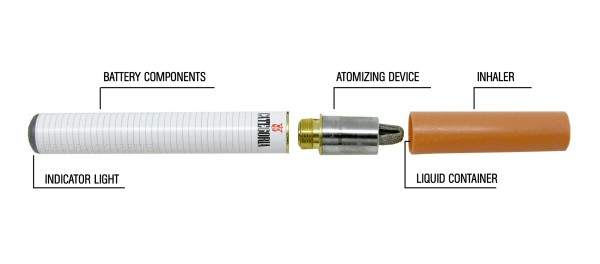
**Structure of the 'Categoria' electronic-cigarette (e-Cigarette)**. The e-Cigarette is a battery-powered electronic nicotine delivery device (ENDD) resembling a cigarette designed for the purpose of providing inhaled doses of nicotine by way of a vaporized solution to the respiratory system. This device provides a flavor and physical sensation similar to that of inhaled tobacco smoke, while no smoke or combustion is actually involved in its operation. It is composed of the following key components: (1) the inhaler - also known as 'cartridge' (a disposable non-refillable plastic mouthpiece - resembling a tobacco cigarette's filter - which contains an absorbent material that is saturated with a liquid solution containing nicotine); (2) the atomizing device (the heating element that vaporizes the liquid in the mouthpiece and generates the mist with each puff); (3) the battery component (the body of the device - resembling a tobacco cigarette - which houses a lithium-ion re-chargeable battery to power the atomizer). The body of the device also houses an electronic airflow sensor to automatically activate the heating element upon inhalation and to light up a red LED indicator to signal activation of the device with each puff. Each pre-filled 'Original' cartridges used in this study contains nicotine (7.25 mg/cartridge) dissolved in propylene glycol (233.7 mg/cartridge) and vegetable glycerin (64.0 mg/cartridge) [details can be found at: http://www.liaf-onlus.org/public/allegati/categoria1b.pdf].

The electronic-cigarette (e-Cigarette) is a battery-powered electronic nicotine delivery device (ENDD) resembling a cigarette designed for the purpose of nicotine delivery,where no tobacco or combustion is necessary for its operation [9] (Figure 1). Consequently, this product may be considered as a lower risk substitute for factory-made cigarettes. In addition, people report buying them to help quit smoking, to reduce cigarette consumption and to relieve tobacco withdrawal symptoms due to workplace smoking restrictions [10]. Besides delivering nicotine, e-Cigarettes may also provide a coping mechanism for conditioned smoking cues by replacing some of the rituals associated with smoking gestures (e.g. hand-to-mouth action of smoking). For this reason, e-Cigarettes may help smokers to remain abstinent during their quit attempt or to reduce cigarette consumption. A recent internet survey on the satisfaction of e-Cigarette use has reported that the device helped in smoking abstinence and improved smoking-related symptoms [11]. Under acute experimental conditions, two marketed electronic cigarette brands suppressed tobacco abstinence symptom ratings without leading to measurable levels of nicotine or CO in the exhaled breath [12]. The e-Cigarette is a very hot topic that has generated considerable global debate with authorities wanting to ban it or at least regulate it. Consequently, a formal demonstration supporting the efficacy and safety of these devices in smoking cessation and/or smoking reduction studies would be of utmost importance.

With this in mind, we designed a prospective proof-of-concept study to monitor possible modifications in the smoking habits of a group of well characterized regular smokers experimenting the most popular marketed e-Cigarette in Italy ('Categoria'; Arbi Group Srl, Milano, Italy) focusing on smoking reduction and smoking abstinence. We also monitored adverse events and measured participants' perception and acceptance of the product.

## Methods

### Participants

Healthy smokers 18-60 years old, smoking ≥ 15 factory-made cigarettes per day (cig/day) for at least the past 10 years and not currently attempting to quit smoking or wishing to do so in the next 30 days were recruited from the local Hospital staff in Catania, Italy. None of the participants reported a history of alcohol and illicit drug use, major depression or other psychiatric conditions. We also excluded subjects who reported recent myocardial infarction, angina pectoris, high blood pressure (BP > 140 mmHg systolic and/or 90 mmHg diastolic), diabetes mellitus, severe allergies, poorly controlled asthma or other airways diseases. The study protocol was discussed with the Chair of the local institutional ERB (Comitato Etico Azienda Vittorio Emanuele) in February 2010. In consideration of the fact that e-cigarette use is a widespread phenomenon in Italy, that many e-cigarette users are enjoying them as consumer goods, that this type of product is not regulated as a drug or a drug device in Italy (end users can buy e-cig almost anywhere - internet, tobacconists, pharmacies, restaurants, and shops), and that only healthy smokers not willing to quit smoking would participate, it was felt that the study fulfilled the criteria of an observational naturalistic investigation and was exempt from the requirement from ethical approval. Participants gave written informed consent prior to participation in the study.

### Study Design and Baseline Measures

**Figure 2:**
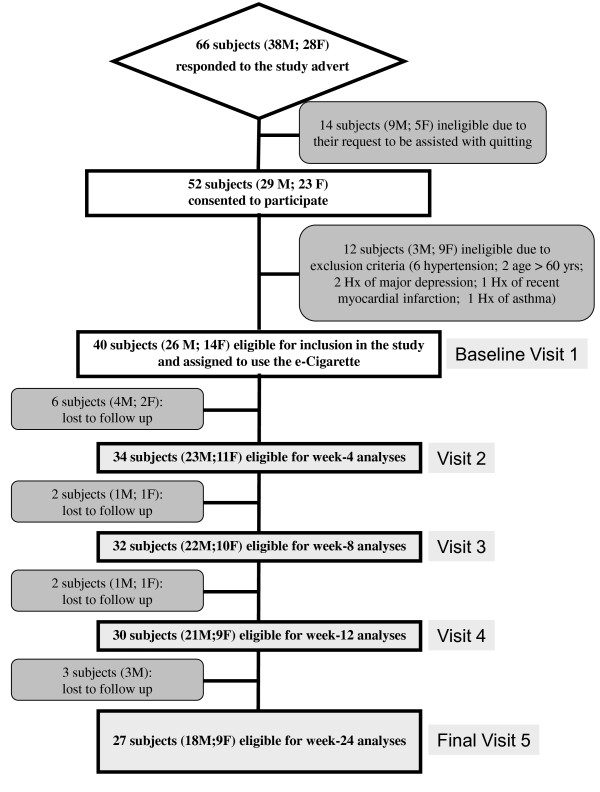
**Number of patients recruited and flow of patients within the study**. A total of 66 subjects with specifically predefined smoking criteria (smoking ≥ 15 cig/day for at least the past 10 years) responded to the advert; of these, 14 subjects were not included in the study because they spontaneously seek assistance with quitting (these were then invited to attend the local smoking cessation clinic, which offers standard support with cessation counselling and pharmacotherapy for nicotine dependence). The remaining 52 subjects consented to participate into the study; of these, 12 were not considered eligible because of the exclusion criteria (6 had a high blood pressure, 2 were older than 60; 2 had a diagnosis of major depression; 1 suffered from recent myocardial infarction; 1 had uncontrolled allergic asthma). In the end, 40 volunteers were included in the study and were issued with e-Cigarette kits loaded with nicotine cartridges. By the end of the study, a total of 13 subjects were lost to follow-up due to failure of attending their control visits. Overall 27 participants were available for analyses at the 24-week follow-up visit.

Eligible participants were invited to use an ENDD ('Categoria' e-Cigarette, Arbi Group Srl, Milano, Italy) and were followed up prospectively for 6 months. They attended a total of five study visits at our smoking cessation clinic (Centro per la Prevenzione e Cura del Tabagismo (CPCT), Università di Catania, Italy): a baseline visit and four follow-up visits, (at week-4, week-8, week-12 and week-24) (Figure 2).

At baseline (study visit 1), basic demographic and a detailed smoking history were taken and individual pack-years (pack/yrs) calculated together with scoring of their level of nicotine dependence by means of Fagerstrom Test of Nicotine Dependence (FTND) questionnaire [13]. Subjective ratings of depression were assessed with the Beck Depression Inventory (BDI) [14]. Additionally, levels of carbon monoxide in exhaled breath (eCO) were measured using a portable device (Micro CO, Micro Medical Ltd, UK). Participants were given a free e-Cigarette kit containing two rechargeable batteries, a charger, and two atomizers and instructed on how to charge, activate and use the e-Cigarette. Key troubleshooting were addressed and phone numbers were supplied for both technical and medical assistance. A full 4-weeks supply of 7.4 mg nicotine cartridges ("Original" cartridges; Arbi Group Srl, Milano, Italy) was also provided and participants were trained on how to load them onto the e-Cigarette's atomizer. Random checks confirmed that the nicotine content per cartridge was 7,25 mg. Detailed toxicology and nicotine content analyses of "Original" cartridges had been carried in a laboratory certified by the Italian Institute of Health and can be found at: http://www.liaf-onlus.org/public/allegati/categoria1b.pdf

Participants were permitted to use the study product *ad libitum *throughout the day (up to a maximum of 4 cartridges per day, as recommended by the manufacturer) in the anticipation of reducing the number of cig/day smoked, and to fill a 4-weeks' study diary recording product use, number of any tobacco cigarettes smoked, and adverse events.

Participants were invited to came back at week-4 (study visit 2), week-8 (study visit 3), and week-12 (visit 4), a) to receive further free supply of nicotine cartridges together with the study diaries for the residual study periods, b) to record their eCO levels, and c) to give back completed study diaries and unused study products.

Study participants attended a final follow-up visit at week-24 (study visit 5) to report product use (cartridges/day) and the number of any tobacco cigarettes smoked (from which smoking reduction and smoking abstinence could be calculated), to re-check eCO levels and to rate the degree of usefulness of the study product. In particular, participants were asked to rate their level of satisfaction with the products compared to their usual cigarettes using a visual analogue scale (VAS) from 0 to 10 points (0 = being 'completely unsatisfied', 10 being = 'fully satisfied'); on the same scale, they also rated helpfulness (in keeping them from smoking) and whether they would recommend it to a friend who wanted to stop/reduce smoking. Adverse events were obtained from their study diaries.

Given the observational nature of this study, no emphasis on encouragement, motivation and reward for the smoking cessation effort were provided since this study was intended to monitor the case of a smoker (unwilling to quit) trying out an unconventional nicotine delivery device in a real world setting. Although participants were allowed to smoke their own brand of cigarette as they wished, smoking cessation services were provided to those who would spontaneously ask for assistance with quitting. These subjects were excluded from the study protocol.

### Study outcome measures

The primary efficacy measure was sustained 50% reduction in the number of cig/day at week-24 from baseline (***reducers***) [15]; defined as sustained self-reported 50% reduction in the number of cig/day compared to baseline for the 30 days period prior to week-24 study visit (eCO levels were measured to verify smoking status and confirm a reduction compared to baseline).

A secondary efficacy measure of the study was sustained 80% reduction in the number of cig/day at week-24 from baseline (***heavy reducers***); defined as sustained self-reported 80% reduction in the number of cig/day compared to baseline for the 30 days period prior to week-24 study visit (eCO levels were measured to verify smoking status and confirm a reduction compared to baseline).

An additional secondary efficacy measure of the study was sustained smoking abstinence at week-24 (***quitters***); defined as complete self-reported abstinence from tobacco smoking (not even a puff) for the 30 days period prior to week-24 study visit (eCO levels were measured to objectively verify smoking status with an eCO concentration of ≤10 ppm).

Those smokers who failed to meet the above criteria at the final week-24 follow-up visit (study visit 5) were categorized as reduction/cessation failures (***failures***).

### Statistical Analyses

This was a proof-of-concept pilot study, the first of its kind, hence no previous data could be used for power calculation. However, using our previous experience in smoking cessation studies, we estimated that a sample of 40 subjects would have been adequate to acquire quit/reduction rates from 70-75% of the subjects enrolled [16]. Primary and secondary outcome measures were computed by including all enrolled participants - assuming that all those individuals who were lost to follow-up are classified as failures (intention-to-treat analysis). The changes from baseline (study visit 1) in number of cig/day and in eCO levels were compared with data recorded at subsequent follow-up visits using Wilcoxon Signed rank test as these data were non-parametric. Parametric and non-parametric data were expressed as mean (± SD) and median (interquartile range (IQR)) respectively. Correlations were calculated using Spearman's Rho Correlation. Statistical methods were 2-tailed, and P values of < 0.05 were considered significant.

## Results

### Participant characteristics

After excluding for the study exclusion criteria, a total of 40 (M 26; F 14; mean (± SD) age of 42.9 (± 8.8) years) regular smokers (mean (± SD) pack/yrs of 34.9 (± 14.7)) consented to participate and were included in the study (Table 1; Figure 2). Twenty-seven (67.5%) completed all study visits and returned for their final follow-up visit at week-24. Baseline characteristics of those who were lost to follow-up were not significantly different from participants who completed the study.

**Table 1:** Patient Demographics

	Parameter	Mean (± SD)*
Subjects eligible for inclusion(n = 40)		
	Age	42.9 (± 8.8)
	Sex	26M; 14F
	Smoking Years	26.9 (± 8.8)
	FTND	6.0 (6, 8)*
	Beck Depression Inventory	9 (5, 12.3)*
	Cigarettes/day	25 (20, 30)*
	eCO	23.5 (15.8, 36)*

†Subjects available for week-24 analyses(n = 27)		
	Age	42.6 (± 8.4)
	Sex	18M; 9F
	Smoking Years	27.2 (± 8.9)
	FTND	7 (6, 7)*
	Beck Depression Inventory	9 (5, 12.5)*
	Cigarettes/day	25 (20, 30)*
	eCO	24 (15.5, 37)*

*Non-parametric data expressed as median (IQR).

† Subjects excluding those lost-to-follow-up.

**Abbreviations**: SD - Standard Deviation; M - Male; F - Female; FTND - Fagerstrom Test of Nicotine Dependence; eCO - exhaled carbon monoxide; IQR - interquartile range.

### Outcome measures

**Figure 3:**
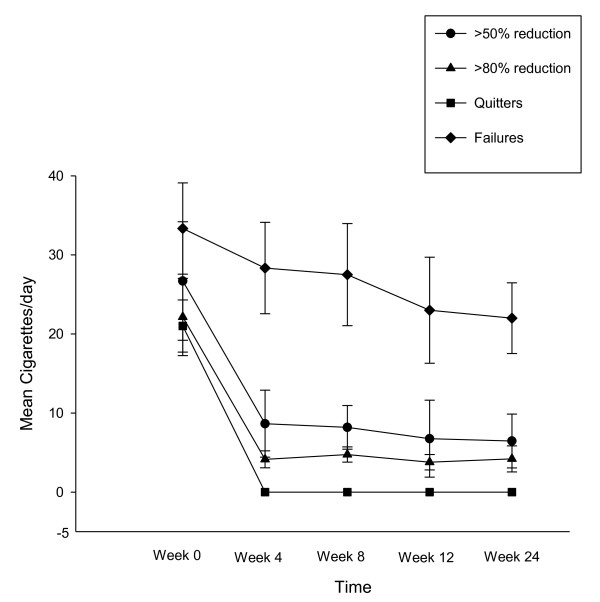
**Changes in the mean (± SD) cigarette use for each study subgroups throughout the study**.

**Figure 4:**
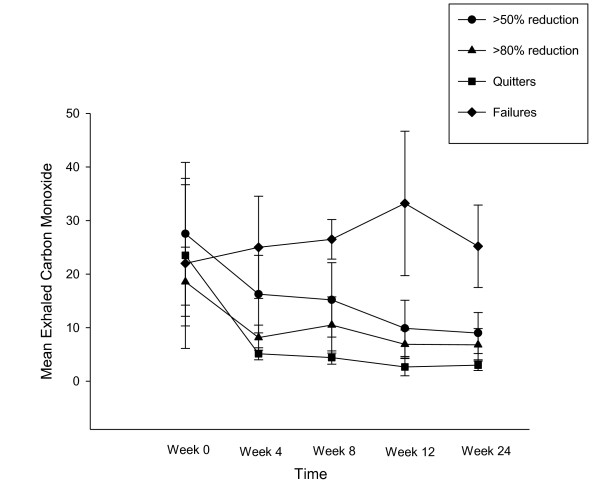
**Changes in the mean (± SD) eCO levels for each study subgroups throughout the study**.

Participants' smoking status at baseline and at 24-week is shown on Table 2. Taking the whole cohort of participants (n = 40), an overall 80% reduction in median cig/day use from 25 to 5 was observed by the end of the study (p < 0.001). Sustained 50% reduction in the number of cig/day at week-24 was shown in 13/40 (32.5%) participants, with a median of 25 cig/day (IQR 20, 30) decreasing significantly to 6 cig/day (IQR 5, 6)(p < 0.001). Of these tobacco smoke reducers, five (12.5%) could be classified as sustained heavy reducers (at least 80% reduction in the number of cig/day) at week-24. They had a median consumption of 30 cig/day (IQR 25, 35) at baseline, decreasing significantly to 3 cig/day (IQR 3, 6) (p = 0.043). There were 9/40 (22.5%) quitters, with 6/9 still using the e-Cigarette by the end of the study. Overall, combined sustained 50% reduction and smoking abstinence was shown in 22/40 (55%) participants, with a median of 25 cig/day (IQR 20, 30) decreasing significantly to 3 cig/day (IQR 0, 6)(p < 0.001), which is equivalent to an overall 88% reduction. Details of mean cigarette use and eCO levels throughout the study is shown in Figure 3 and 4.

**Table 2:** Subject Parameter Outcomes Following 24 Weeks of Electronic Cigarette Use

Parameter	AT BASELINE	AT 24-Weeks Post E-Cigarette	*p value‡*
Sustained 50% (excluding quitters) reduction in cigarette smoking (n = 13)			
Age	40.1 (± 7.7)†	6 (5, 6)*	< 0.001
Sex	8M; 5F	8 (6, 11)*	0.001
Smoking Years	24.5 (± 8.7)†		
Cigarettes/day	25 (20, 30)*		
eCO	18 (14, 33)*		

Sustained 80% (excluding quitters) reduction in cigarette smoking (n = 5)			
Age	40.6 (± 10.4)†	3 (3, 6)*	0.043
Sex	4M; 1F	6 (4, 10)*	0.042
Smoking Years	25.4 (± 11.8)†		
Cigarettes/day	30(25, 35)*		
eCO	15 (14, 44)*		

Sustained 100% (quitters) reduction in cigarette smoking (n = 9)			
Age	44.7 (± 9.3)†	0 (0, 0)*	0.008
Sex	8M; 1F	3 (2, 3)*	0.008
Smoking Years	29 (± 9.6)†		
Cigarettes/day	25 (23, 30)*		
eCO	31 (23, 41)*		

Sustained > 50% (including quitters) reduction in cigarette smoking (n = 22)			
Age	42 (± 8.5)†	3 (0, 6)*	< 0.001
Sex	16M; 6F	5.5 (3, 9.5)*	< 0.001
Smoking Years	26.3 (± 9.1)†		
Cigarettes/day	25 (20, 30)*		
eCO	27 (15.5, 37.5)*		

Smoking Failure (< 50% smoking reduction) (n = 5)			
Age	45.6 (± 7.9)†	20 (20, 20)*	0.157
Sex	2M; 3F	28 (17, 31)*	0.892
Smoking Years	31.2 (± 7)†		
Cigarettes/day	25 (20, 25)*		
eCO	18 (16, 32)*		

**Abbreviations**: SD - Standard Deviation; M - Male; F - Female; eCO - exhaled carbon monoxide.

‡p value - within group Wilcoxon Signed Rank Test.

† Parametric data expressed as mean (± SD).

*Non-parametric data expressed as median (interquartile range(IQR)).

### Product Use

**Figure 5:**
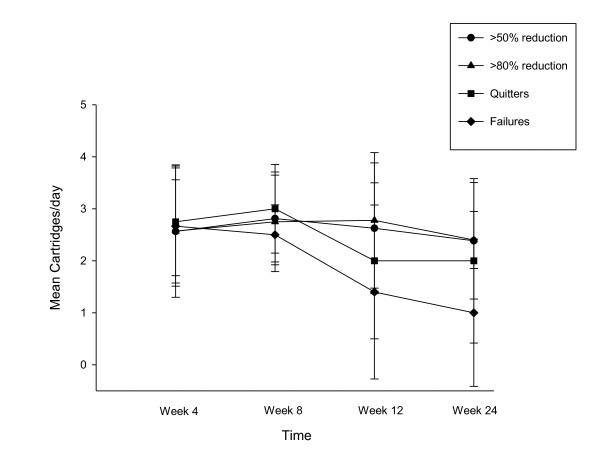
**Changes in the mean (± SD) cartridge use for each study subgroups throughout the study**.

Details of mean cartridge use throughout the study is shown in Figure 5. The reported number of cartridges/day used by our study participants was dissimilar, ranging from a maximum of 4 cartridges/day (as per manufacturer's recommendation) to a minimum of 0 cartridges/day ('zero' was recorded in the study diary, when the same cartridge was used for more than 24 hours). For the whole group (n = 27), a mean (± SD) 2.0 (± 1.4) cartridges/day was used throughout the study. The number of cartridges/day used was slightly higher when these summary statistics were computed with the exclusion of the eight study failures; the value increasing to a mean (± SD) of 2.2 (± 1.3) cartridges/day. Correlation between the number of cartridges/day and smoking reduction in those participants with sustained 50% reduction in smoking was not significant (Rho -0.003; p = 0.988). Likewise, the correlation between the number of cartridges/day, and combined sustained 50% reduction and smoking abstinence was also non-significant (Rho -0.185; p = 0.546).

### Adverse Events

The most frequently reported adverse events were mouth irritation (20,6%), throat irritation (32,4%), and dry cough (32,4%) (Table 3). These events were most commonly reported at the beginning of the study and appeared to wane spontaneously by study visit 5. Remarkably, side effects commonly recorded during smoking cessation trials with drugs for nicotine dependence were absent (i.e. depression, anxiety, insomnia, irritability, hunger, constipation were not reported). Moreover, no serious adverse events (i.e. events requiring unscheduled visit to the family practitioner or hospitalisation) occurred during the study.

**Table 3:** Adverse events reported by participants who completed all study visits

Adverse Event	Study Visits			
	
	4-week n/n (%)	8-week n/n (%)	12-week n/n (%)	24-week n/n (%)
Throat irritation*	11/34 (32,4%)	5/32 (15,6%)	5/30 (16,7%)	4/27 (14,8%)

Mouth Irritation*	7/34 (20,6%)	4/32 (12,5%)	3/30 (10,0%)	2/27 (7,4%)

Sore Throat	4/34 (11,8%)	1/32 (3,1%)	1/30 (3,3%)	0/27 (0,0%)

Dry cough	11/34 (32,4%)	6/32 (18,8%)	3/30 (10,0%)	3/27 (11,1%)

Dry mouth	3/34 (8,8%)	1/32 (3,1%)	1/30 (3,3%)	1/27 (3,7%)

Mouth ulcers	1/34 (2,9%)	1/32 (3,1%)	1/30 (3,3%)	0/27 (0,0%)

Dizziness^§^	5/34 (14,7%)	2/32 (6,3%)	2/30 (6,7%)	1/27 (3,7%)

Headache	4/34 (11,8%)	2/32 (6,3%)	2/30 (6,7%)	1/27 (3,7%)

Nausea	5/34 (14,7%)	2/32 (6,3%)	1/30 (3,3%)	1/27 (3,7%)

* Throat and mouth irritation were described either as tickling, itching, or burning sensation

^§ ^Dizziness, was also used to mean vertigo and light-headedness.

### Product Preferences

The 'Categoria' e-Cigarette rated scores well above the mean for satisfaction and for helpfulness (enabling them to refrain from smoking), their mean (± SD) VAS values being 6.3 (± 2.5) and 7.5 (± 2.7) respectively. Moreover, it was observed that participants would enthusiastically recommend the e-Cigarette to friends or relatives who wanted to stop/reduce smoking, the mean (± SD) VAS value being 8.0 (± 3.4). Predictably, the e-Cigarette rated even higher scores when these summary statistics were computed with the exclusion of the study failures (n = 8). On the contrary, the perception and acceptance of the product by those who failed to remain abstinent or to reduce smoking (n = 5) was poor; the mean (± SD) VAS values for satisfaction and for helpfulness being 2.2 (± 0.8) and 2.5 (± 1.0), respectively. As expected, these individuals were unlikely to recommend the 'Categoria' e-Cigarette to friends or relatives; the mean (± SD) VAS value being 2.3 (± 1.2).

Among the most positive features of e-Cigarettes were the pleasure of inhalation and exhalation of the vapour. Other positive features mentioned included cleaner and fresher breath, absence of odours in clothing and hair. Although the overall participants' perception and acceptance of the product was good, its ease of use could be improved and technical defects reduced. During the course of the study, five study participants could not use the product as recommended and had to be retrained within 72 hours. Three participants reported that the device often failed to produce mist when puffed (three atomizers had to be substituted). Another two were given a faulty charger (chargers were immediately replaced). According to study participants, perception and acceptance of the product could be improved by increasing manufacturing standards, by providing a recharge lasting at least 24 hours, by reducing the weight of the device and by substituting the hard plastic mouthpiece.

## Discussion

In this pilot study, we have shown for the first time that substantial and objective modifications in the smoking habits may occur in smokers using e-Cigarettes, with significant smoking reduction and smoking abstinence and no apparent increase in withdrawal symptoms. Participants were not only enthusiastic about using the e-Cigarette, but the majority (67.5%) were also able to adhere to the program and to return for the final follow-up visit at week-24 with an overall quit rate of 22.5%. Moreover, at least 50% reduction in cigarette smoking was observed in 32.5% of participants. Overall, combined reduction and smoking abstinence was shown in 55% of participants. These preliminary findings are of great significance in view of the fact that all smokers in the study were, by inclusion criteria, not interested in quitting. Although not directly comparable with classic cessation and/or reduction studies with other pharmaceutical products because of its design (the present study is not an ordinary cessation study), the results of our study are in general accordance with the findings published in the medical literature [17].

The large magnitude of this effect suggests the e-Cigarette strongly suppressed cigarette use. However, no correlations were observed between the number of nicotine cartridges/day used and the level of smoking reduction. This is not unexpected, in view of the powerful interaction between physical and behavioural dependence of smoking [18,19] and the modest increases in blood nicotine levels measured after the use of this type of devices [20]. Therefore, it is unlikely that the observed positive effect of the e-Cigarette is due to nicotine delivery. Rather, the strong suppression of smoking in association with the absence of correlation between cartridges use and level of smoking reduction, suggests that the positive effect of the e-Cigarette may be also due to its capacity to provide a coping mechanism for conditioned smoking cues by replacing some of the rituals associated with smoking gestures (e.g. hand-to-mouth action of smoking). In agreement with this, we have recently demonstrated that nicotine free inhalators can only improve quit rates in those smokers for whom handling and manipulation of their cigarette played an important role in their ritual of smoking [21].

Although dry cough and mouth ulcers can be associated with withdrawal effects, typical withdrawal symptoms of smoking cessation trials with drugs for nicotine dependence were not reported during the course of the study. It is possible that the e-Cigarette by providing a coping mechanism for conditioned smoking cues could mitigate withdrawal symptoms associated with smoking reduction and smoking abstinence. In contrast from other ENDDs such as Eclipse (which is known to generate substantial level of eCO) [22], e-Cigarettes use does not lead to increased eCO levels [12]. In the present study, the smoking reduction with 'Categoria' e-Cigarette use was associated to a substantial decrease in the level of eCO. The most frequent adverse events were mouth irritation, throat irritation and dry cough, but all appeared to wane spontaneously with time. These are likely to be secondary to exposure to propylene glycol mist generated by the e-Cigarette's atomizer. Propylene glycol is a low toxicity compound widely used as a food additive and in pharmaceutical preparations. Exposure to propylene glycol mist may occur from smoke generators in discotheques, theatres, and aviation emergency training and is known to cause ocular, mouth, throat, upper airway irritation and cough [23,24]. Dizziness was often reported by participants at the beginning of the study and can be brought about by the hyperventilation associated to the greater puffing time with the e-Cigarette. Alternatively, the dizziness as well as other reported adverse events such as nausea and headaches may be due to nicotine overuse. The substantial reduction in the frequency of dizziness observed by the end of the study may be due to the improved familiarisation with the puffing technique and/or to the overall reduction in nicotine use. Therefore, the 'Categoria'" e-Cigarette can be seen as a safe way to smoke although larger and longer studies will be required for a full assessment of its adverse events.

The 'Categoria' e-Cigarette rated high scores for a range of subjective ratings of user preferences suggesting that the product was functioning as an adequate cigarette substitute. Hence, participants were more likely to recommend the e-Cigarette to friends or relatives. Conversely, as would be expected the perception and acceptance of the product by those who failed to remain abstinent or to reduce smoking was poor and these individuals were unlikely to recommend the e-Cigarette. We cannot exclude that technical problems (particularly those who went unreported) and difficulty of use (it takes time to familiarize with the puffing technique) could have affected the number of lost to follow-up and failures. Although the overall participants' perception and acceptance of the product was good, its ease of use could be improved. Technical defects could be reduced by increasing manufacturing standards, providing a recharge lasting at least 24 hours, reducing the weight of the device and substituting the hard plastic mouthpiece. These latter two suggestions would improve device acceptability for certain common rituals of cigarette smoking, e.g. keeping the cigarette between lips.

Harm-reduction strategies are aimed at reducing the adverse health effects of tobacco use in individuals unable or unwilling to quit. Reducing the number of cig/day is one of several kinds of harm reduction strategies [25]. It is uncertain whether substantial smoking reduction in smokers using the e-Cigarette will translate in health benefits, but a number of studies have analyzed the ability of smoking reduction to lower health risks and have reported some reductions in cardiovascular risk factors and lung cancer mortality [26-28]. Moreover, reduction in cigarette smoking by e-Cigarette may well increase motivation to quit as indicated by a substantial body of evidence showing that gradually cutting down smoking can increase subsequent smoking cessation among smokers [15,29-32]. While not the treatment of choice, reduced smoking strategies might be considered for recalcitrant smokers unwilling to quit, as in the case of our study population.

There are some limitations in our study. Firstly, this was a small uncontrolled study, hence the results observed may be due to a chance finding and not to a true effect; consequently the results should be interpreted with caution. However, it would have been quite problematic to have a placebo arm in such a study. Secondly, 32.5% of the participants failed to attend their final follow-up visit, but this is not unexpected in a smoking cessation study. Thirdly, because of its unusual design (smokers not willing to quit, e-Cigarettes were used throughout the entire study period) this is not an ordinary cessation study and therefore direct comparison with other smoking cessation products cannot be made. Fourthly, failure to complete the study and smoking cessation failures could be due to occurrence of technical defects for the e-Cigarette. However, this could not be assessed with confidence in the present study. Lastly, assessment of withdrawal symptoms in our study was not rigorous. Withdrawal was assessed at each visit by simply asking about the presence/absence of irritability, restlessness, difficulty concentrating, increased appetite/weight gain, depression or insomnia. It is likely that this way of collecting information is liable to recall bias. Therefore, the reported lack of withdrawal symptoms in the study participants should be considered with caution.

## Conclusions

Current smoking cessation interventions can increase the chance of quitting in committed smokers who are already motivated and prepared to stop smoking [33], but a broader range of interventions are needed in order to bring more smokers into treatment and increase the numbers who are motivated to make quit attempts. Although not formally regulated as a pharmaceutical product, the e-Cigarette can help smokers to remain abstinent or reduce their cigarette consumption. By replacing tobacco cigarettes, the e-cigarette can only save lives.

Here we show for the first time that e-Cigarettes can substantially decrease cigarette consumption without causing significant side effects in smokers not intending to quit. However, large and carefully conducted RCTs will be required before a definite answer about the efficacy and safety of these devices can be formulated. Some of these trials are now in progress in Italy [34-36] and New Zealand [37] and hopefully they will be able to confirm and expand the preliminary observations reported in the present article.

## Abbreviations

e-Cigarette: Electronic-Cigarette; ENDD: Electronic Nicotine Delivery Device; Cig/day: Cigarettes smoked per day; BP: Blood pressure; mmHg: millimetres of mercury; FTND: Fagerstrom Test of Nicotine Dependence; BDI: Beck's Depression Inventory; eCO: exhaled carbon monoxide; mg: milligrams; Cartridges/day: cartridges used per day; VAS: Visual Analogue Score; ppm: parts per million; Pack/yrs: pack-years; SD: standard deviation; IQR: interquartile range

## Competing interests

None of the authors have any competing interests to declare, but RP has received lecture fees from Pfizer and, from Feb 2011, he has been serving as a consultant for Arbi Group Srl.Arbi Group Srl (Milano, Italy), the manufacturer of the e-Cigarette supplied the product, and unrestricted technical and customer support. They were not involved in the study design, running of the study or analysis and presentation of the data.

## Authors' contributions

RP: Principal investigator, protocol design, interpretation of the data, writing of the ms; PC: conduction of the study, interpretation of the data, writing of the ms; JBM: statistical analyses, interpretation of the data, writing of the ms; GP: recruiting of patients, conduction of the study, writing of the ms; DC: recruiting of patients, conduction of the study; CR: protocol design, interpretation of the data, writing of the ms. All authors have read and approved the final manuscript.

## Pre-publication history

The pre-publication history for this paper can be accessed here:

http://www.biomedcentral.com/1471-2458/11/786/prepub
